# Postoperative Delirium and Neurocognitive Disorders: A Comprehensive Review of Pathophysiology, Risk Factors, and Management Strategies

**DOI:** 10.7759/cureus.68492

**Published:** 2024-09-02

**Authors:** Sharayu Paunikar, Vivek Chakole

**Affiliations:** 1 Anesthesiology, Jawaharlal Nehru Medical College, Datta Meghe Institute of Higher Education and Research, Wardha, IND

**Keywords:** cognitive impairment, surgery, management strategies, risk factors, pathophysiology, neurocognitive disorders, postoperative delirium

## Abstract

Postoperative delirium (POD) and neurocognitive disorders (NCDs) are common and serious complications that can occur after surgery, particularly in older adults and those with preexisting cognitive impairments. These conditions are associated with significant morbidity, increased healthcare costs, and reduced quality of life. Understanding the underlying mechanisms, risk factors, and effective management strategies for POD and NCDs is critical for improving patient outcomes and reducing the burden on healthcare systems. This comprehensive review aims to synthesize current knowledge on the pathophysiology, risk factors, and management strategies for POD and NCDs. It explores the neurobiological and molecular mechanisms contributing to these conditions, identifies the patient-related, surgical, and environmental factors that increase risk, and evaluates pharmacological and non-pharmacological approaches to prevention and treatment. A thorough literature review was conducted using recent studies, clinical guidelines, and expert consensus to provide a detailed overview of the pathophysiology, risk factors, clinical presentation, prevention, and management of POD and NCDs. The pathophysiology of POD and NCDs involves complex interactions between neuroinflammatory processes, neurotransmitter imbalances, and brain network disruptions. Risk factors include advanced age, preexisting cognitive impairment, type and duration of surgery, and perioperative complications. Management strategies emphasize a multidisciplinary approach, incorporating preoperative optimization, careful intraoperative management, and postoperative interventions. Pharmacological treatments, such as antipsychotics, and non-pharmacological approaches, including environmental modifications and cognitive rehabilitation, play crucial roles in management. Postoperative delirium and NCDs are multifactorial conditions with significant impacts on surgical outcomes. Effective management requires a comprehensive understanding of their pathophysiology and risk factors and the implementation of targeted prevention and treatment strategies. Future research should focus on personalized approaches to prevention and treatment, further elucidation of mechanisms, and developing predictive models to enhance care for patients at risk of these neurocognitive complications.

## Introduction and background

Postoperative delirium (POD) and neurocognitive disorders (NCDs) are significant complications that can arise following surgery, particularly among older adults and those with pre-existing cognitive impairment. POD is characterized by an acute and fluctuating disturbance in attention, awareness, and cognition that typically develops within hours to days after surgery [1]. Neurocognitive disorders, including postoperative cognitive dysfunction (POCD), encompass a broader range of cognitive impairments that may develop after surgery and persist for weeks, months, or even years. These conditions pose a challenge to patient care and have profound implications for patient outcomes, healthcare costs, and quality of life [2].

Understanding the mechanisms and factors contributing to POD and NCDs is crucial for improving surgical outcomes and enhancing postoperative recovery [3]. The prevalence of POD ranges widely depending on the type of surgery, patient demographics, and the criteria used for diagnosis, with rates reported as high as 50% in certain populations, such as those undergoing cardiac or orthopedic surgeries [4]. Similarly, NCDs can have a lasting impact, with some patients experiencing persistent cognitive deficits that affect their ability to return to normal life activities. These disorders are associated with increased morbidity and mortality, longer hospital stays, higher rates of discharge to long-term care facilities, and substantial economic burden [5].

Given the significant impact of POD and NCDs on both patients and healthcare systems, it is essential to develop a comprehensive understanding of their pathophysiology, risk factors, and management strategies [6]. This review aims to thoroughly examine the current knowledge on POD and NCDs, including their underlying biological mechanisms, the risk factors that predispose certain individuals to these conditions, and the latest strategies for prevention and management. By integrating insights from recent research, clinical guidelines, and expert consensus, this review seeks to enhance the understanding and management of these complex postoperative complications, ultimately improving patient care and outcomes.

## Review

Definition and classification

Postoperative delirium and NCDs, such as postoperative cognitive dysfunction (POCD), are significant complications that can follow surgical procedures. Postoperative delirium is defined as an acute onset of fluctuating changes in mental status, marked by disturbances in attention, awareness, and cognition occurring after surgery [6]. This condition can develop within hours to days postoperatively, especially in older adults, with incidence rates varying from 5% to 52% based on factors such as the type of surgery and patient vulnerabilities [7]. In contrast, NCDs encompass a wider range of cognitive impairments, with POCD specifically referring to cognitive decline resulting from surgical interventions. POCD is characterized by persistent memory, attention, and executive function deficits beyond the immediate postoperative period [7]. Postoperative delirium can be classified into three main subtypes, each with distinct clinical features. Hyperactive delirium is characterized by restlessness, agitation, and hypervigilance and may include hallucinations or delusions. This subtype is often easily identified due to the heightened activity levels of the patient [8]. In contrast, hypoactive delirium, the most common form, presents with lethargy, reduced motor activity, and a slow response to stimuli. This subtype is frequently underdiagnosed because of its subtle presentation, despite potentially having worse prognoses. Mixed delirium features elements of both hyperactive and hypoactive delirium, with patients fluctuating between agitation and lethargy [9].

Neurocognitive disorders encompass a range of conditions, with POCD being a specific type linked to surgical procedures. Other NCDs include dementia, a chronic and progressive condition characterized by significant cognitive decline that interferes with daily life. Delirium, often acute and transient, can occur in various medical settings beyond postoperative scenarios. Understanding these distinctions is crucial for accurate diagnosis and management [10]. Diagnosis of POD and POCD involves several criteria and assessment tools. According to The Diagnostic and Statistical Manual of Mental Disorders, Fifth Edition (DSM-5), delirium is diagnosed based on criteria that include a disturbance in attention and awareness that develops over a short period (usually hours to days) and fluctuates in severity. This disturbance must be a direct physiological consequence of another medical condition, substance intoxication, withdrawal, or exposure to toxins [11]. The Confusion Assessment Method (CAM) is widely used for diagnosing delirium, focusing on acute onset, inattention, disorganized thinking, and altered levels of consciousness. The Mini-Mental State Examination (MMSE) is a brief 30-point questionnaire used to screen for cognitive impairment and assess various cognitive domains, including orientation, memory, and attention. More comprehensive neuropsychological testing may be conducted to evaluate cognitive function and identify specific deficits associated with POCD [12].

Epidemiology

Postoperative delirium and NCDs are critical concerns in surgical populations, particularly among older adults [13]. The incidence of POD varies widely across different surgical settings, with reported rates ranging from 5% to 52% in older adults following noncardiac surgery. Systematic reviews suggest a pooled incidence of approximately 18.4% [14]. Rates can be higher in specific surgical contexts; for example, POD incidence ranges from 11% to 46% in cardiac surgeries and from 13% to 50% in noncardiac surgeries. In orthopedic procedures, the incidence can reach 12% to 51%. Acute-care surgical populations have even higher rates, ranging from 18% to 55%, compared to elective surgeries [15]. Several demographic factors influence the incidence of POD and NCDs. Age is a major risk factor and the likelihood of developing POD increases. For every 15-year increment beyond 60 years, the rates of POD nearly double, making older adults, especially those aged 75 and above, the most vulnerable [15]. Preexisting cognitive impairments, such as dementia, further elevate this risk. Gender also plays a role, with some studies indicating that men may be slightly more at risk for POD, although findings are inconsistent and require further research. Comorbidities, including frailty and other neuropsychiatric disorders, are significant risk factors for both POD and long-term cognitive dysfunction [16]. The consequences of POD and NCDs extend well beyond the immediate postoperative period and have significant implications for patient outcomes. Mortality rates are notably higher among patients who experience POD, with studies showing a twofold increase in one-year mortality rates. Additionally, patients with POD often face prolonged hospital stays, increased days of mechanical ventilation, and extended admissions to the intensive care unit (ICU) [16]. Research estimates that delirium can lead to a 2.5-fold increase in one-year medical costs due to complications associated with delirium. Long-term cognitive impairment is another severe consequence, with a notable correlation between POD and cognitive decline. Studies suggest that delirium can result in a 2.8-fold increase in the rate of cognitive decline over three years post-surgery, and the risk of developing dementia may increase tenfold in patients who experience postoperative delirium [17].

Pathophysiology

Neurobiological Mechanisms

The neurobiological mechanisms underlying POD are complex and involve several processes that contribute to cognitive dysfunction. One key mechanism is inflammation and neuroinflammation. Surgical procedures initiate a systemic inflammatory response marked by the release of pro-inflammatory cytokines such as interleukin-1 (IL-1), IL-6, and tumor necrosis factor-alpha (TNF-α) [18]. These cytokines mediate the body’s response to injury and can significantly impact brain function. Once released, they can enter the central nervous system (CNS) and activate microglia, the brain’s resident immune cells. Activated microglia release further inflammatory mediators, sustaining a cycle of neuroinflammation that can disrupt neuronal function and connectivity [19]. Another crucial aspect of neuroinflammation is disrupting the blood-brain barrier (BBB). The inflammatory response can increase BBB permeability, which protects the brain from harmful substances. Inflammatory cytokines can compromise the integrity of tight junctions between endothelial cells, allowing neurotoxic substances, immune cells, and cytokines to enter the CNS [19]. This disruption can result in neuronal injury and contribute to the acute cognitive changes observed in POD. The presence of inflammatory mediators in the brain can impair synaptic plasticity and neurotransmission, further aggravating cognitive dysfunction [20]. Neurotransmitter imbalances also significantly contribute to the development of POD. Changes in dopamine levels can impact cognitive processes. Increased dopamine activity in certain brain regions may lead to hyperactivity and agitation, while decreased activity in others can result in cognitive impairment and reduced attention. The balance of dopamine is essential for maintaining cognitive function, and disruptions can manifest as symptoms of delirium, including altered mood and attention deficits [20]. Additionally, acetylcholine is critical in attention, memory, and learning. The cholinergic hypothesis of delirium posits that an acetylcholine deficiency contributes to cognitive impairments. Surgical stress and anesthesia can further diminish acetylcholine levels, exacerbating this deficiency. Reduced cholinergic activity can impair attention and memory recall, hallmark features of delirium [21]. Oxidative stress and mitochondrial dysfunction are also key contributors to the pathophysiology of POD. Surgical procedures can lead to increased production of reactive oxygen species (ROS), harmful byproducts of cellular metabolism. Elevated ROS levels can cause oxidative damage to proteins, lipids, and DNA, resulting in cellular dysfunction. In the context of POD, oxidative stress can impair neuronal function and viability, contributing to cognitive decline [22]. Additionally, oxidative stress is associated with neuroinflammation, creating a vicious cycle that exacerbates cognitive impairment. Mitochondria, essential for energy production in neurons, can be adversely affected by surgical stress. Impaired mitochondrial function can lead to reduced ATP production and increased ROS generation. This dysfunction results in energy deficits in neurons, making them more vulnerable to injury and contributing to cognitive dysfunction. Moreover, impaired mitochondrial function can trigger apoptosis (programmed cell death), further impacting brain health [23].

Genetic and Molecular Factors

The apolipoprotein E (APOE) gene, especially the ε4 allele, is a well-established genetic risk factor for Alzheimer's disease (AD) and other NCDs. The APOE gene encodes three major isoforms-APOE2, APOE3, and APOE4-that differ in their amino acid sequence at positions 112 and 158 [23]. The APOE4 allele is found in approximately 25%-30% of the general population and about 40% of individuals with late-onset Alzheimer's disease (LOAD). Those who inherit one copy of the APOE4 allele face an increased risk of developing AD, which is even higher for individuals with two copies. The presence of the APOE4 allele is associated with an earlier onset of AD symptoms, potentially occurring 10-15 years earlier per allele. APOE4 carriers also exhibit increased brain amyloid deposition and dysfunction in the medial temporal lobe, including the hippocampus, which is critical for memory and cognitive functions [24]. In addition to AD, APOE4 is a risk factor for other neurocognitive disorders, such as dementia with Lewy bodies (DLB). The mechanism by which APOE4 contributes to DLB is thought to involve disruption in the transport of the protein alpha-synuclein, leading to its accumulation in the brain and resulting in impaired neuronal function [25]. While the role of epigenetic changes in the pathogenesis of delirium and neurocognitive disorders is less extensively studied, emerging evidence suggests that these modifications may be significant. Altered DNA methylation patterns have been observed in the brains of individuals with AD, potentially contributing to the dysregulation of genes involved in the disease process [26]. Histone modifications, such as acetylation and methylation, are also implicated in AD pathogenesis and may affect the expression of genes related to neuroinflammation and synaptic function. Additionally, altered microRNA (miRNA) expression has been noted in the brains of individuals with AD and other NCDs, indicating that these small, non-coding RNAs might regulate gene expression and develop these disorders [26].

Brain Network Changes

Postoperative delirium and postoperative neurocognitive disorders (PND) are associated with alterations in brain network connectivity and structure, which may contribute to the cognitive impairments observed in these conditions [27]. Postoperative delirium has been linked to reduced functional connectivity strength within brain networks, a change that persists even after the symptoms of delirium have resolved. This indicates that delirium may have enduring effects on brain network function. Patients who experience POD exhibit decreased functional connectivity compared to those who do not develop delirium, and this diminished connectivity is associated with significant cognitive decline in the months following surgery. During episodes of delirium, resting-state functional MRI (fMRI) studies have shown network disintegration, with reduced connectivity between brain regions. These changes in functional connectivity may underlie the acute cognitive disturbances observed during delirium [28]. While some studies have not found a significant association between brain atrophy or white matter hyperintensities and the incidence of POD, other research suggests that preexisting structural brain changes may increase susceptibility to delirium following surgery. Presurgical diffusion MRI has identified neural substrates that may predispose individuals to postoperative delirium, indicating that certain structural connectivity patterns could make some patients more vulnerable to developing delirium after surgery [29]. Additionally, delirium has been linked to long-term cognitive decline, with the severity of delirium correlating with the extent of brain network disruption. This suggests that the structural and functional brain changes associated with delirium may contribute to the development of persistent NCDs [29].

Risk Factors

**Figure 1:**
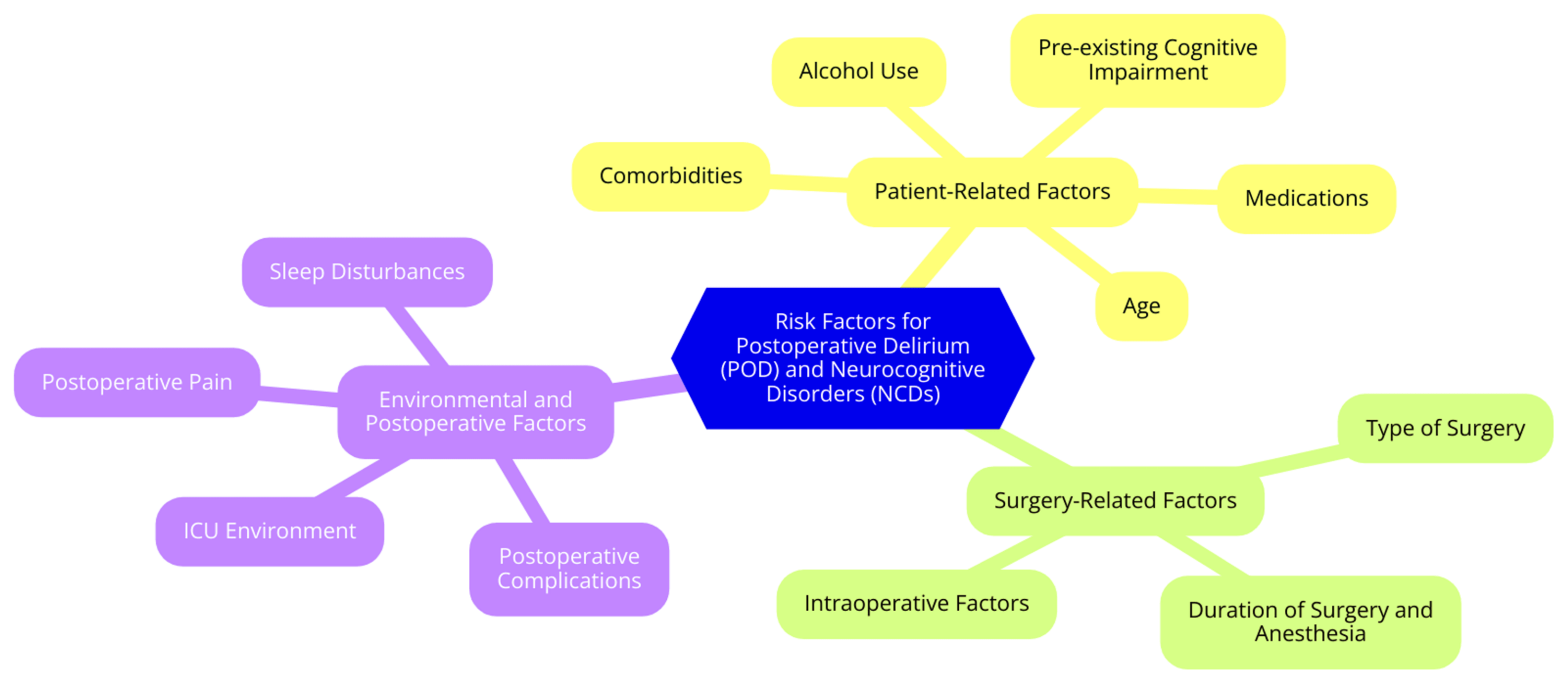
Risk factors for postoperative delirium (POD) and neurocognitive disorders (NCDs) Image Credit: Dr Sharayu Paunikar

Understanding the risk factors associated with POD and POCD is crucial for effective prevention and management. These risk factors can be categorized into three main groups: patient-related factors, surgery-related factors, and environmental and postoperative factors [30]. Patient-related factors significantly influence the development of POD and POCD. Age is a key risk factor; older adults are particularly vulnerable due to age-related physiological changes and cognitive decline. Individuals with preexisting cognitive impairments, such as mild cognitive impairment or dementia, are also at a higher risk for these complications [31]. Comorbidities further increase this risk. Conditions like cardiovascular disease can affect cerebral perfusion, while diabetes, particularly with poor glycemic control, can cause microvascular complications impacting cognitive function. Depression is another significant factor, as a history of depression is associated with increased susceptibility to cognitive decline after surgery [32]. Medications also play a role in the risk profile for POD and POCD. Anticholinergic medications can impair cognitive function, raising the likelihood of delirium. Similarly, benzodiazepines, especially in elderly patients, can cause sedation and confusion, further increasing the risk of postoperative cognitive disturbances [33]. Surgery-related factors are critical in evaluating the risk of POD and POCD. The type of surgery performed can significantly impact outcomes; for instance, cardiac surgery is often associated with a high incidence of POD due to factors such as cardiopulmonary bypass and prolonged anesthesia. Orthopedic surgeries, particularly those involving major joints, can also lead to notable postoperative cognitive changes. The duration of surgery and anesthesia is another important consideration; longer procedures and extended exposure to anesthesia are linked to a higher risk of cognitive dysfunction due to increased physiological stress and the potential for complications [34]. Intraoperative factors such as hypoxia, hypotension, and blood loss can contribute to cognitive impairment. Hypoxia, or reduced oxygen levels during surgery, can negatively affect brain function, while hypotension can lead to inadequate cerebral perfusion. Significant blood loss can result in hypovolemia, further increasing the risk of cognitive dysfunction [35]. Environmental and postoperative factors can also significantly affect the development of POD and POCD. Ineffective postoperative pain management can contribute to delirium, making effective pain control strategies essential for cognitive recovery. The ICU environment also plays a role; noise, lighting, and frequent monitoring can disrupt sleep patterns, increasing the risk of delirium [36]. Additionally, postoperative infections and complications, such as pneumonia or urinary tract infections, can exacerbate delirium and cognitive decline. Monitoring and managing these complications is crucial for optimal recovery and minimizing cognitive disturbances [36]. Risk factors for POD and NCDs are illustrated in Figure 1.

Clinical Presentation and Diagnosis

Postoperative delirium is marked by an acute alteration in mental status, which can include confusion, agitation, drowsiness, impaired attention, disorganized speech, and, in severe cases, delusions and hallucinations. Postoperative NCDs encompass a broader spectrum of cognitive impairments. This includes POCD, which is characterized by persistent cognitive decline following surgery, and long-term cognitive decline associated with both POD and POCD, potentially advancing to dementia in some instances [36]. Distinguishing between delirium, dementia, and depression is essential. Delirium is characterized by its acute onset, fluctuating course, and reversibility, often resolving by treating underlying causes. In contrast, dementia is marked by a gradual onset, progressive decline, and irreversibility, typically associated with neurodegenerative diseases. Depression may present with cognitive symptoms, but these are often linked to a lack of motivation rather than genuine cognitive deficits and can improve with the treatment of mood disturbances [37]. Diagnostic criteria and tools are critical for identifying and managing POD and NCDs. The DSM-5 criteria for delirium require evidence of disturbances in attention and awareness, along with a change in cognition that a preexisting condition cannot explain. Screening tools such as the CAM and the Delirium Rating Scale are useful for identifying and assessing the severity of delirium. At the same time, neuropsychological testing is employed to diagnose POCD and evaluate various cognitive domains post-surgery [38]. Understanding the timing and progression of cognitive symptoms after surgery is crucial. Delirium may manifest within hours to days following surgery, often peaking around the first or second postoperative day. The risk of POCD is notably higher in the first month, particularly in patients who experienced POD during their hospital stay. Although POD is generally transient, it can lead to longer-term cognitive decline, especially in patients with preexisting vulnerabilities such as mild cognitive impairment or dementia [20].

Prevention strategies

Preoperative Interventions

Preoperative interventions are vital in mitigating the risk of POD and POCD. Among these interventions, cognitive rehabilitation programs have emerged as a promising strategy. These structured programs aim to bolster cognitive resilience before surgery by engaging patients in activities designed to enhance mental agility [39]. Key components of cognitive prehabilitation include memory tasks and problem-solving exercises that sharpen cognitive function. Additionally, integrating physical activity supports overall health and well-being, positively impacting cognitive performance. Mindfulness and stress reduction techniques, such as meditation and deep breathing exercises, are also crucial, as they alleviate anxiety and stress that could worsen cognitive decline. Educating patients about the surgical process and potential risks empowers them, reduces anxiety, and improves coping strategies, leading to better postoperative outcomes [39]. Medication optimization is another essential preoperative intervention focused on minimizing the risk of cognitive complications. This strategy involves a comprehensive review of a patient’s medication regimen to identify and adjust any medications that may pose a risk. Reducing polypharmacy, especially among older adults who often take multiple medications, is critical. By assessing the necessity of each medication and discontinuing those that are non-essential or potentially harmful, healthcare providers can significantly decrease the likelihood of adverse cognitive effects [40]. Additionally, avoiding high-risk drugs such as benzodiazepines, anticholinergics, and certain opioids is crucial, as these medications can impair cognitive function and increase the risk of delirium. Optimizing medication use enhances overall safety and improves patient outcomes, reducing the potential for postoperative cognitive decline [41]. Preoperative counseling and risk assessment are also fundamental to a comprehensive preoperative strategy. This involves thoroughly evaluating the patient’s health status, including cognitive function, medical history, and psychosocial factors, to identify individual risk factors for POD and POCD. Educating patients about the surgical procedure, expected outcomes, and potential complications is essential for setting realistic expectations and reducing anxiety [42]. Involving family members in counseling ensures they understand the patient’s needs and can provide support during recovery. Collaboratively setting recovery goals empowers patients and enhances their motivation to engage in postoperative rehabilitation activities. This holistic approach increases patient awareness and preparedness and facilitates early identification of high-risk individuals, allowing for targeted interventions to significantly improve postoperative outcomes [43].

Intraoperative Strategies

Intraoperative strategies are vital in reducing the risk of POD and POCD. Whenever possible, regional anesthesia techniques such as spinal, epidural, or peripheral nerve blocks should be considered over general anesthesia, as they are associated with lower rates of POD and POCD [44]. Minimizing the dose and duration of anesthetic agents, particularly in older patients, can help mitigate their cognitive impact. It is important to carefully titrate anesthesia to achieve the necessary depth while avoiding oversedation. Combining analgesic methods, such as regional anesthesia with non-opioid medications, can offer effective pain control while limiting opioid use, which may contribute to delirium [44]. Close monitoring of vital signs such as blood pressure, heart rate, and oxygen saturation is crucial for promptly identifying and addressing any hemodynamic instability. Preventing hypothermia is essential, as it has been linked to an increased risk of POD and POCD. Ensuring adequate hydration and avoiding both hypovolemia and hypervolemia helps maintain hemodynamic stability and proper organ perfusion. Strategies to keep blood pressure within acceptable ranges, such as using vasopressors or adjusting the depth of anesthesia, should be employed, as prolonged or severe hypotension during surgery is associated with a higher risk of POD and POCD [44]. Maintaining adequate oxygenation throughout the procedure is critical to prevent hypoxia, which can adversely affect cognitive function. Keeping blood glucose levels within the normal range during surgery may also reduce the risk of POD and POCD. Techniques to minimize surgical stress, such as opting for minimally invasive procedures or reducing surgical duration, help mitigate the inflammatory response and its potential impact on cognitive function [45]. Effective intraoperative management requires close collaboration among anesthesiologists, surgeons, and other healthcare professionals. Regular communication, adherence to best practices, and ongoing quality improvement initiatives are key to optimizing intraoperative care and reducing the incidence of postoperative neurocognitive disorders [46]. Although no single intraoperative approach can eliminate the risk of POD and POCD, combining evidence-based techniques tailored to each patient’s needs can significantly improve outcomes. Continued research and the implementation of best practices are essential to enhancing perioperative care and minimizing the burden of postoperative neurocognitive complications [46].

Postoperative Management

Postoperative management is crucial in minimizing complications such as POD and POCD. Effective strategies promote recovery, enhance cognitive function, and ensure patient comfort. Key components of postoperative care include early mobilization and rehabilitation, pain management, and non-pharmacological interventions [2]. Early mobilization is vital for preventing complications and supporting cognitive benefits. Initiating physical activity shortly after surgery helps mitigate the risk of deep vein thrombosis (DVT), pneumonia, and muscle atrophy. Furthermore, early mobilization has been shown to improve cognitive function and reduce the incidence of delirium. To implement this strategy effectively, healthcare providers should assess each patient’s physical capabilities and readiness for mobilization as soon as possible post-surgery [47]. Patients can start with passive range-of-motion exercises and progressively move to sitting, standing, and walking as tolerated. A multidisciplinary approach involving physical, occupational, and nursing staff is essential for developing personalized rehabilitation plans. Encouraging and supporting patients during mobilization helps build confidence and reduce anxiety, thereby facilitating recovery [48]. Pain management is another critical element of postoperative care. A multimodal analgesia approach, which combines various medications and techniques, can provide optimal pain control while minimizing side effects. This strategy not only enhances pain management but also reduces dependence on opioids, thereby decreasing the risk of opioid-related side effects such as sedation and delirium [48]. Effective pain management includes the use of non-opioid analgesics (e.g., acetaminophen, non-steroidal anti-inflammatory drugs (NSAIDs)), regional anesthesia (e.g., nerve blocks), and adjuvant medications (e.g., gabapentinoids). Regular pain assessments using standardized scales enable healthcare providers to adjust the pain management plan. Additionally, it is important to limit the use of high-risk medications, such as benzodiazepines and certain opioids, which can worsen delirium and cognitive dysfunction [49]. Non-pharmacological interventions are also essential in postoperative care. Reorientation techniques can help address confusion and disorientation, common in POD. Frequent patient communication, including reminders about the date, time, and location, can significantly aid reorientation. Visual aids, such as clocks, calendars, and signage, can further assist patients in orienting themselves. Involving family members in reorientation efforts provides familiar faces and voices that offer comfort and reassurance during recovery [50]. Promoting good sleep hygiene is also crucial for cognitive recovery and overall well-being. Creating a quiet, dark, and comfortable environment encourages restful sleep. Establishing a consistent bedtime routine, including relaxation techniques such as deep breathing or calming music, can enhance sleep quality. Minimizing nighttime disruptions by clustering care activities and reducing night noise also improves sleep outcomes [51].

Management of postoperative delirium and neurocognitive disorders

Pharmacological Management

Pharmacological management of POD should only be considered after non-pharmacological interventions have been attempted. The primary objectives of pharmacotherapy are to manage agitation, reduce the risk of harm, and alleviate distressing symptoms. However, evidence supporting specific pharmacological agents is limited, requiring a careful and individualized approach [51]. Antipsychotics are the most commonly employed pharmacological agents for managing POD. Haloperidol is the most extensively studied antipsychotic for this purpose. It can be administered orally, intramuscularly, or intravenously, with initial dosing typically starting at low levels (0.25-0.5 mg every four hours) for elderly patients. Higher doses may be required for persistent agitation. Atypical antipsychotics, such as olanzapine, are also used, though evidence comparing their efficacy to haloperidol is mixed. Some studies indicate that olanzapine may not offer significant advantages over haloperidol for managing POD. Antipsychotics should be used cautiously due to potential side effects, including prolonged QT interval and extrapyramidal symptoms. Patients on antipsychotics require vigilant monitoring, including ECG assessments [52]. Other pharmacological agents have been explored for their potential role in managing POD. Cholinesterase inhibitors, such as rivastigmine, have been investigated but are not routinely recommended for treating POD based on current evidence. Conversely, dexmedetomidine, an alpha-2 agonist, shows promise in preventing and treating POD, particularly in agitated patients. However, further research is needed to clarify its definitive role. Benzodiazepines are generally avoided due to their risk of exacerbating delirium unless used specifically for alcohol or benzodiazepine withdrawal [53]. The risks and benefits of pharmacological interventions must be carefully evaluated. While these medications can manage severe agitation and reduce the risk of harm, they do not address the underlying causes of delirium. Additionally, antipsychotics and other sedatives may worsen cognitive impairment and increase the risk of falls, especially in older adults. Therefore, the potential benefits of pharmacotherapy should be weighed against the risks on an individual patient basis. It is crucial to use pharmacotherapy judiciously, employing the lowest effective dose for the shortest duration possible [54].

Non-pharmacological Approaches

Non-pharmacological approaches are essential in managing POD and NCDs. These strategies focus on creating a supportive environment and enhancing cognitive function without relying on medications. Key interventions include cognitive and behavioral strategies, environmental modifications, and family involvement [54]. Cognitive and behavioral interventions aim to improve cognitive function and reduce confusion. Engaging patients in simple cognitive activities, such as puzzles, word searches, or card games, can stimulate mental activity and provide a sense of accomplishment. Regular reorientation techniques like reminding patients of their surroundings, the date, and the purpose of their hospitalization ground them in reality [55]. Establishing a consistent daily routine is also beneficial, reducing anxiety and confusion by providing predictability. Teaching relaxation techniques, such as deep breathing exercises or guided imagery, can alleviate anxiety and enhance overall well-being. Positive reinforcement can manage agitation or restlessness by encouraging calmness and reassurance, thus creating a more stable environment [55]. Creating a supportive physical environment is crucial for minimizing the risk of delirium. Modifications such as maximizing natural light exposure during the day can regulate circadian rhythms, improving sleep quality and cognitive function [56]. Adjustable lighting to reduce glare and provide softer illumination in the evening fosters a calming atmosphere conducive to rest. Reducing unnecessary noise from medical equipment, alarms, and conversations is also important. Utilizing sound-absorbing materials or white noise machines can mask disruptive sounds and promote a more peaceful setting. Allowing patients to have personal items, such as photographs or favorite blankets, provides comfort and a sense of security, helping to alleviate feelings of disorientation [56]. Involving family members in the care process enhances patient comfort and cognitive function. Educating family members about delirium, its signs, and how they can assist empowers them to participate actively in the patient’s care. A calm presence and reassurance from family members can significantly benefit the patient. Encouraging family visits helps reduce feelings of isolation and anxiety, and flexible visitation policies that allow family members to be present enhance emotional support [57]. Additionally, involving family members in daily care activities, such as feeding or assisting with mobility, fosters a sense of connection and purpose for the patient and the family. Tailoring care strategies to meet individual needs and preferences improves the patient’s overall experience and respects their routines, preferences, and cultural backgrounds [58].

Multidisciplinary Care Models

Multidisciplinary care models for managing POD and NCDs emphasize the importance of close collaboration among various healthcare professionals to achieve optimal patient outcomes. Two key components are the involvement of geriatric consultation teams and the coordination between surgeons, anesthesiologists, and nurses [59]. Geriatric consultation teams are vital in managing POD and neurocognitive disorders, particularly in older surgical patients. Preoperatively, these teams assess older adults for cognitive impairment, frailty, and other risk factors, enabling targeted interventions to mitigate risks [60]. Postoperatively, geriatric specialists offer guidance on managing delirium, optimizing pain control, promoting mobility, and addressing other age-related syndromes. They work closely with physical and occupational therapists to facilitate rehabilitation and coordinate with social workers and caregivers to ensure a safe discharge plan. Additionally, geriatric specialists monitor for persistent cognitive deficits and provide long-term support to enhance functional recovery [60]. Effective coordination between surgeons, anesthesiologists, and nurses is crucial for preventing and managing POD and neurocognitive disorders. Preoperatively, the team collaborates to discuss the surgical plan, anesthetic approach, and strategies to minimize delirium risk. Intraoperatively, anesthesiologists carefully adjust medications to prevent over-sedation and ensure adequate organ perfusion, while surgeons perform procedures efficiently to reduce physiological stress [61]. Nurses play a key role by monitoring for signs of delirium and intervening promptly. During postoperative handoff, clear communication ensures continuity of care, emphasizing relevant risk factors and management plans. Daily interdisciplinary rounding allows for coordinated assessment and management of delirium, with nurses monitoring mental status and updating the team on any changes. Additionally, nurses and other team members involve families in reorientating, stimulating, and supporting patients with delirium, as families provide valuable collateral information [62].

Long-Term Outcomes and Prognosis

Postoperative delirium and its long-term outcomes are significant concerns in surgical care, especially among older adults. The effects of POD extend beyond the immediate postoperative period, impacting cognitive function and quality of life and increasing the risk of recurrent delirium or progression to dementia. Research has shown that POD is associated with substantial long-term cognitive decline [63]. Patients who experience POD often exhibit a greater decline in activities of daily living (ADL) and face an increased risk of mortality within 24 to 36 months following surgery. For example, a study revealed that individuals who developed POD had a more significant decline in ADL scores (16 ± 15 vs. 9 ± 15) and a higher mortality rate (29% vs. 9%) compared to those without POD. Furthermore, delirium has been linked to a 2.8-fold increase in the rate of cognitive decline over three years, indicating that the cognitive impairments associated with POD can persist long after the initial episode. As assessed by health-related quality-of-life indices, quality of life also deteriorates in patients with POD, particularly in physical function and social engagement [64]. The recovery trajectory for patients who experience POD can be complex and prolonged. Many older adults face delayed or incomplete cognitive recovery, complicating their overall rehabilitation process. Studies indicate that patients with POD often require more extensive rehabilitation services and may experience prolonged hospital stays, resulting in increased healthcare costs and a greater burden on caregivers. Effective rehabilitation strategies must address cognitive and functional impairments, emphasizing early mobilization, cognitive exercises, and supportive care to enhance recovery outcomes [64]. Patients with a history of POD are at heightened risk of recurrent delirium. Preexisting cognitive impairments and other comorbidities exacerbate this risk. There is also evidence suggesting that POD may act as a precursor to more severe cognitive disorders, including dementia. Ongoing research is exploring the relationship between POD and subsequent dementia, with some studies indicating that patients who experience delirium may be at an increased risk of developing dementia later in life [65].

Future directions and research

Future research into POD and NCD is crucial for advancing patient care. Key areas for exploration include addressing current knowledge gaps, investigating emerging therapies, and evaluating personalized medicine approaches [65]. A major gap in our understanding is the long-term impact of POD on cognitive function. Although evidence links POD to long-term cognitive decline, the precise mechanisms and nature of this relationship are not fully understood. Further research is needed to elucidate how POD affects cognitive outcomes over extended periods, especially beyond three months post surgery [66]. Additionally, existing prevention strategies for POD have variable effectiveness, and their impact on long-term cognitive health remains uncertain. Future studies should aim to identify effective interventions that address both POD and its enduring effects on cognitive function. There is also a need for standardized assessment tools and protocols for diagnosing POD and POCD. Diagnostic criteria and assessment method variations complicate comparisons across studies and impede the development of cohesive management strategies [66]. Emerging therapies and interventions offer promising opportunities to improve outcomes for patients at risk of POD and NCD. Pharmacological approaches, including the evaluation of antipsychotics and anti-inflammatory agents, could open new avenues for treatment. Research should assess the efficacy and safety of these therapies across diverse surgical populations. Non-pharmacological interventions, such as cognitive training, environmental modifications, and programs like the Hospital Elder Life Program (HELP), have shown promise in reducing POD rates [67]. Future research should focus on the scalability and effectiveness of these strategies in different clinical settings. Additionally, the impact of various anesthetic agents and techniques on the incidence of POD and POCD warrants further investigation. Research should explore how different anesthetic depths and types affect cognitive outcomes post-surgery [67]. Personalized medicine approaches represent an exciting frontier in managing POD and NCD. Genetic and biomarker research could help identify individuals at higher risk for these conditions. Biomarkers related to inflammation, neurodegeneration, or cognitive resilience might inform tailored interventions. Developing models incorporating patient-specific factors such as age, preexisting cognitive impairment, and comorbidities could improve risk stratification and enable targeted preventive measures. Personalized treatment strategies, including customized rehabilitation programs and targeted cognitive therapies, could enhance outcomes for patients at risk of POD and NCD. Future research should evaluate the effectiveness of these personalized approaches in clinical practice [68].

## Conclusions

Postoperative delirium and NCDs represent significant challenges in perioperative care, with profound implications for patient outcomes, healthcare costs, and quality of life. These conditions, which range from acute and fluctuating episodes of delirium to long-term cognitive dysfunction, are influenced by a complex interplay of patient-related, surgical, and environmental factors. Understanding the pathophysiology of POD and NCDs, including the roles of neuroinflammation, neurotransmitter imbalances, and genetic predispositions, is critical for developing targeted prevention and management strategies. Current approaches emphasize a multidisciplinary strategy that includes preoperative optimization, intraoperative vigilance, and postoperative management to minimize risk and promote recovery. Despite advances in our understanding and management of POD and NCDs, gaps remain in our knowledge, particularly regarding individualized treatment approaches and the long-term outcomes of affected patients. Future research should focus on elucidating the underlying mechanisms further, developing predictive tools, and refining therapeutic strategies to better prevent and manage these conditions. By addressing these gaps, we can improve patient care and enhance recovery trajectories for surgical patients at risk of neurocognitive complications.
